# P02-13 Perceived health and impact of a physical activity intervention in sex-offenders and other inmates

**DOI:** 10.1093/eurpub/ckac095.032

**Published:** 2022-08-29

**Authors:** Daniela Anastasi, Simone Digennaro, Marica Ciccarelli, Bruno Federico

**Affiliations:** Department of Human Sciences, Society and Health, University of Cassino and Southern Lazio, Cassino, Italy

**Keywords:** prison, sex-offenders, physical activity

## Abstract

**Background:**

Prisoners spend most of their time in restricted environments, with strong limitations on freedom and social relationships. Inmates that committed sex crimes (sex-offenders) are subject to limitations more restrictive than other inmates. Physical inactivity is very common in these populations, with negative effects on both physical and mental health. Interventions that contrast physical inactivity are strongly encouraged in this context.

**Methods:**

A health promotion intervention was carried out in the prison of Cassino (central Italy) in 2019. This intervention aimed to promote prisoners' health and physical activity, and increase their life skills. Intervention sessions, which included body awareness and proprioceptive exercises, were carried out two times a week for about 9 months. Focus group interviews were carried out with 9 sex-offenders and, separately, with 20 other inmates, exploring the topics of self-perceived health, and impact of the health promotion intervention.

**Results:**

Both groups reported feelings of psychological stress and anxiety, leading to increases in cigarette smoking and excessive food consumption. Several focus group participants felt the need to do physical activity on a regular basis and they asked for sport facilities and programs. Sex-offenders reported that the health promotion intervention helped them fill the time, enjoy and relax, while non sex-offenders reported an increase in physical fitness and viewed this intervention as an opportunity of social rehabilitation.

**Conclusions:**

Imprisonment leads to negative health behaviours, such as physical inactivity, cigarette smoking, and excessive food consumption, particularly among sex-offenders that are subject to stronger limitations than other inmates. Interventions that promote physical activity among prisoners may have positive effects on prisoners' physical fitness and mental health.

